# A case of organophosphate poisoning presenting with seizure and unavailable history of parenteral suicide attempt

**DOI:** 10.4103/0974-2700.76825

**Published:** 2011

**Authors:** Vinay Pandit, Shubha Seshadri, S N Rao, Charmaine Samarasinghe, Ashwini Kumar, Rohith Valsalan

**Affiliations:** Department of Medicine, Kasturba Medical College, Manipal, Manipal University, Karnataka, India; 1 Department of Neurology, Kasturba Medical College, Manipal, Manipal University, Karnataka, India; 2 Department of Community Medicine, Kasturba Medical College, Manipal, Manipal University, Karnataka, India

**Keywords:** Organophosphate poison, parenteral, seizure

## Abstract

Organophosphate (OP) poisoning is common in India. Only few case reports of parenteral OP poisoning have been described. We report a case of self-injected methyl parathion poisoning, presenting after four days with seizure, altered sensorium, and respiratory distress which posed a diagnostic and therapeutic dilemma. Despite nonavailability of history of OP poisoning, he was treated based on suspicion and showed a good clinical response to treatment trial with atropine and pralidoxime, and had a successful recovery. Atypical presentations may be encountered following parenteral administration of OP poison, and even a slight suspicion of this warrants proper investigations and treatment for a favorable outcome. Persistently low plasma cholinesterase level is a useful marker for making the diagnosis.

## INTRODUCTION

Organophosphate (OP) poisoning is common in developing countries and especially so in India. Poisoning occurs mostly by voluntary ingestion, inhalation, or by absorption through the skin. Toxicity can also occur rarely by self injection through intramuscular or intravenous route. OP poisoning by parenteral route has been described by very few authors.[1–6] If the history of parenteral administration of the compound is not available, diagnosis becomes difficult. OP poisoning by parenteral route may manifest acutely with cholinergic crisis and respiratory distress, intermediate syndrome, or with delayed toxicity. We describe a case of self injected methyl parathion, presenting with seizure and abscess in the arm, pulmonary edema, and flaccid quadriparesis, which was successfully treated on clinical judgment.

## CASE REPORT

A male student, aged 20 years, was admitted in neuro ICU with an episode of seizure and altered sensorium. He had no premorbid illness. He had travelled to Mumbai four days before admission. Relatives denied consumption of any poison and medications. At the time of hospitalization, he was restless and was in postictal state. Vital signs revealed pulse rate of 62/minute, blood pressure of 120/80 mmHg, respiratory rate of 14 per minute, afebrile, and had plenty of oral secretions. Neurological examination revealed GCS of 6/15 with reduced movements of all four limbs. Pupils were pin point bilaterally with absent Doll’s eye movement. Plantar reflex was extensor bilaterally. Deep tendon reflexes were sluggish. There were no fasciculation and no smell of OP compound. He had cellulitis of left arm. Examination of chest showed bilateral crepitations. Examination of other systems was normal. Investigations at admission showed normal renal functions, liver functions, and normal serum levels of sodium, potassium, calcium, and magnesium. Blood picture showed leukocytosis. Chest X-ray showed bilateral haziness suggesting acute respiratory distress syndrome. Ultrasonography of left arm showed pus collection in the intramuscular plane. Debridement was done and 250 ml of pus was drained. At this point of time, differential diagnosis of metabolic encephalopathy, toxic encephalopathy due to sepsis, possible brain stem diseases, and OP poisoning/drug over dosage were considered. Computed tomography and magnetic resonance imaging scan of the brain, lumbar puncture and CSF analysis were done and they were normal. His EKG, cardiac enzymes, and echocardiography were normal and blood, urine, and pus cultures were sterile. Screening for benzodiazepine, antiepileptic drugs were negative. Serum cholinesterase level was 1234 units (reference range- 5000 – 9000 units). On day 2, he developed respiratory distress with carbon dioxide retention, ABG revealed PaCO_2_ of 54 mmHg, and he required ventilator support. At this point of time, we had reasonably excluded metabolic and structural causes for his problem; hence, possibility of OP poisoning was considered on the basis of respiratory failure, pulmonary secretions, supported by low plasma cholinesterase level. Ryle’s tube aspiration was done at the time of hospitalization and gastric aspirate was minimal. Empirically, he was treated with atropine and pralidoxime along with broad spectrum antibiotics. Atropine was given 5 mg bolus, followed by infusion at the rate of 2 mg/h, and the dose was titrated as per his clinical response and signs of atropinisation. Response to atropine treatment was good and over five days gradual improvement in sensorium was noticed. Pralidoxime was given at a dose of 1 gm infusion, three times per day for initial two days. He was treated with phenytoin sodium for seizures. Initial antibiotics were piperacillin-tazobactam and metronidazole but during the course of illness, there were worsening of chest shadows and antibiotics were changed to meropenem and linezolid. Cultures of endotracheal tube secretions were sterile. His chest X-ray and oxygenation improved. In the initial three to four days, fluctuation in the sensorium was noticed but continued to have neuroparalysis, neck muscle weakness, and his respiratory efforts were poor. His restlessness was controlled with diazepam. He continued to require ventilator support for breathing. We kept talking to relatives regarding possible consumption of OP poison, but they had no clue about any such event. Plasma cholinesterase level was repeated and value had gone down to 934 units. His restlessness was better, became more alert and neuroparalysis started recovering slowly. The entire problem got sorted out on sixth day, when he communicated to us in writing that he had injected metacid (methyl parathion) to his left arm while travelling in train. He required ventilator support for 12 days and recovered completely. He revealed that he had injected poison with suicidal intention and all the legal protocols were done as per the hospital rules. Following recovery, he was evaluated by psychiatrists and revealed that injection of poison was an impulsive act due to poor social and financial support from family.

## DISCUSSION

India is a predominantly agrarian country with large rural population. OP pesticides are used commonly for suicidal purpose. Although ingestion with suicidal intent is a common mode, occupational exposure while spraying in fields is an important modality of poisoning. The clinical presentations and outcome of OP poisoning depend not only on the pesticide but also on the dose, the route of administration, and the time between poisoning and start of treatment. The clinical features of OP poising are as follows: (i) acute cholinergic crisis, which manifests within 24 to 72 hours due to accumulation of acetylcholine at muscarinic and nicotinic sites and accumulation in CNS leading to headache, giddiness, seizure, and altered sensorium; (ii) intermediate syndrome, which manifests after 24 to 96 hours due to prolonged activity of acetyl choline at nicotinic receptors resulting in weakness of ocular, neck, limb, and respiratory muscles. The diagnosis of OP poisoning is made from history of ingestion or mucocutaneous exposure, clinical features, and plasma cholinesterase levels. The depressed plasma cholinesterase levels confirm the diagnosis of OP poisoning and the levels continue to be depressed for 4 to 7 weeks. The estimation of red blood cell cholinesterase is more specific. In cases of ingestion of OP compound, gastric lavage is done and sample is collected for analysis and for medico legal purposes. Atropine acts as physiological antidote as it antagonizes muscarinic receptor-mediated actions. Atropine is given as the initial loading dose of 2 to 5 mg and repeated every 5 to 10 minutes until signs of atropinisation appear. After this, it is given as infusion at the rate of 0.02 to 0.08 mg/kg/min and the dose is titrated as per the clinical response.[7,8] Role and dose of oximes are controversial. Pralidoxime is generally used in dose of 1 gm every 6 to 8 hours; recent studies have shown better outcome with high-dose infusion, 18 to 24 gm/day.[9] OP-induced seizure is treated with diazepam. Legal issues are of concern while managing these kinds of cases. While dealing with the cases of suspected poisoning, stomach wash, excreta, and other articles like empty bottle capsules or liquids should be collected and preserved. We registered this case in medico legal registry and notification was sent to the police.[10]

The toxicity of OP poison depends on rapidity with which it gets absorbed to systemic circulation. If the OP compound is administered through parenteral route, absorption and systemic manifestations vary in accordance with plane of administration. Few authors have reported the development of acute cholinergic crisis within 30 minutes of IV administration.[1,2,4] With self injection, symptoms will appear after some delay and if the quantity administered is less, there may be only local abscess. The case we described presented after four days of injection. He had developed abscess in the arm, which may be related to usage of contaminated material. Pus culture was sterile. Possibility of sepsis was considered; however, it was difficult to explain his flaccid quadriperesis, pulmonary edema only on the basis of sepsis, and he showed good response to treatment with atropine. Local site abscess formation is also reported by Nishioka.[3] Local inflammatory findings are to be expected in cases of subcutaneous or intramuscular injection of insecticides. Such injuries are also a potential portal of entry to various organisms. Local debridement is required for drainage of abscess and may have role in clearance of pesticide, if done early.

In all the so far published cases of parenteral OP poisoning, history of injection of the compound was available. The case we described posed a significant dilemma in the diagnosis, as history of injection of the compound was not available at the beginning of treatment. The patient could not provide history because he was in altered sensorium. OP poisoning presenting with seizure is rare and development of seizure following parenteral administration has not been reported yet.

Literature search revealed few cases of parenteral OP poisoning. Badhe and Sudhakar[1] described IV monocrotophos poisoning resulting in intermediate syndrome requiring ventilator support. Raina *et al*.[2] described two cases of dichlorvos poisoning treated with atropine and pralidoxime. Nishioka reported two cases, of which one died because of respiratory failure and the other had only local reaction without systemic toxicity. Guven *et al*.[4] reported an IV methamidophos poisoning which developed features of acute cholinergic crisis within 30 minutes. Zoppellari *et al*.[5] reported a case of injected isofenphos by intramuscular route and developed cholinergic crisis 5 hours after injection, and the signs and symptoms lasted for 3 weeks. The compound which he had injected is commercially marked as metacid-methyl parathion. These are esters of phosphoric acid and methyl parathion is an aryl phosphate. Metacid is the most commonly used and most toxic OP compound in south Asian countries. Fatal dose of this compound is 80 mg by intramuscular route and 175 mg by oral administration.

## CONCLUSION

OP compound toxicity by parenteral route is a diagnostic challenge. Onset of symptoms may be delayed and presentations may be atypical. Even though the symptoms are mild initially, observation for longer period is required. As there are no decontaminating measures, even a small quantity of injection may be fatal. The treating physicians should be vigilant, and appropriate treatment has to be administered in the event of suspicion of OP poison.
